# Editorial Department

**Published:** 1858-11

**Authors:** Lyman Congden

**Affiliations:** Jackson, Tompkins Co., N. Y.


Dr. Flint:

Dear Sir,—I send you the following case, which, if thought to be of sufficient importance, you are at liberty to publish:

*April* 16, 1858, a lad, aged 15, fell asleep lying upon the damp ground, and at the expiration of two or three hours, awoke chilled and with a sore throat. On the evening of the next day I was called, and found the lad suffering from a sensation of impending suffocation, caused by extensive inflammation of the tonsils and all the glands about the mouth, and from his peculiar and difficult breathing and loss of voice, I judged extensive inflammation of the larynx and trachea. It was with much difficulty that I could separate the teeth two lines. The period for depletion had passed. Hoping to accomplish something by the revulsive effect of an active cathartic, I prescribed calomel, grs. xxx, followed by an infusion of senna and jallap, to be administered as speedily as possible, which was very slow in consequence of great difficulty in deglutition; sinapisms to the throat. At the expiration of four hours, the bowels were freely evacuated, much to the relief of the patient.

Prescribed calomel, grs. ii, opium, gr. i, every three hours, and antimony, grs. ii—℥, a teaspoonful every half hour.

*April* 17, morning. The patient much relieved of the distressing pain c[illegible] suffocation.

*April* 18. I was called in great haste, the patient supposed to be dying. On my arrival I was a little surprised to find my patient conversing with ease compared with previous day, and his general aspect much improved. The cause of the alarm I found to be, that the patient was thought to be strangling to death, and whilst strangling he had thrown off a perfect cast of the trachea, measuring four inches in length, and very thick and firm. It being carelessly laid down, the house dog seized and speedily swallowed it, probably supposing it to be his dinner. I lanced both tonsils, which discharged pus freely, and on the following day (my patient being a robust lad) he was walking about the farm.

LYMAN CONGDEN.

JACKSON, Tompkins Co., N. Y., Sept. 4, 1858.

